# Early surgical explantation of a TAVI valve for severe hemolytic anemia caused by mild paravalvular leak

**DOI:** 10.1186/s44215-025-00218-1

**Published:** 2025-07-28

**Authors:** Reo Sakakura, Masazumi Fukuzawa, Hirotaro Sugiyama, Kazuhiro Tani, Taiji Yoshida, Arata Murakami, Hidenobu Terai, Katsushi Ueyama

**Affiliations:** 1 Department of Cardiovascular Surgery, Kanazawa Cardiovascular Hospital, 16, Ha, Tanakamachi, Kanazawa-shi, Ishikawa, 920-0007 Japan; 2 Department of Cardiology, Kanazawa Cardiovascular Hospital, 16, Ha, Tanakamachi, Kanazawa-shi, Ishikawa, 920-0007 Japan

**Keywords:** TAVI, Aortic stenosis, Paravalvular leak, Hemolytic anemia, Valve explantation

## Abstract

**Background:**

Hemolytic anemia following transcatheter aortic valve implantation (TAVI) is an uncommon complication, particularly in patients with only mild paravalvular leak (PVL). Although reports of TAVI valve explantation are increasing, the procedure remains technically demanding and is associated with high morbidity and mortality. This case highlights the importance of early surgical consideration when hemolytic anemia occurs post-TAVI, despite only mild PVL.

**Case presentation:**

An 83-year-old man with symptomatic severe aortic stenosis underwent transfemoral TAVI via a 26-mm SAPIEN 3 Ultra RESILIA® valve. Although classified as low surgical risk, TAVI was chosen on the basis of patient preference and age. The procedure was uneventful, with only mild PVL observed via transesophageal echocardiography. The patient was discharged on postoperative day 10. One month later, he presented with fatigue and laboratory findings indicating severe hemolytic anemia. Echocardiography revealed a PVL jet from the non-coronary to left coronary cusp commissure. Preoperative CT revealed bulky annular calcification, especially at the NCC–LCC commissure. Owing to worsening anemia and ongoing hemolysis, surgical explantation and aortic valve replacement were performed. Intraoperatively, a gap was found between the valve and the annulus at the calcified commissure. The TAVI valve was successfully explanted and replaced with a surgical bioprosthesis. Postoperative recovery was uneventful, and hemolysis resolved completely.

**Conclusions:**

This case demonstrates that mild PVL after TAVI may still cause clinically significant hemolysis depending on anatomical features. Careful preprocedural assessment of annular calcification and commissural geometry is critical, even in low-risk patients. Surgical explantation should be considered early when hemolysis occurs, as delayed intervention may lead to increased morbidity. This case reinforces the need for individualized valve selection and close follow-up to address the adverse hemodynamic consequences of PVL promptly, regardless of its apparent severity.

## Background

Transcatheter aortic valve implantation (TAVI) has become an established minimally invasive treatment for aortic stenosis, especially after being reimbursed in Japan in 2013. With the expansion of indications to include patients at low surgical risk, the number of procedures has continued to rise [1]. Despite its increasing use, complications such as prosthetic valve degeneration, paravalvular leak (PVL), and prosthetic valve endocarditis have emerged, sometimes necessitating surgical explantation of the transcatheter valve [2, 3]. Although PVL is relatively common after TAVI, it is often clinically insignificant when mild. However, in rare instances, even mild leaks can, in certain anatomical or procedural contexts, lead to significant hemolysis [4–7]. Surgical explantation for post-TAVI hemolytic anemia remains infrequently reported because of its technical challenges and associated risks [8]. We report a rare case of early surgical explantation and surgical aortic valve replacement (SAVR) in a low-risk 83-year-old patient who developed severe hemolytic anemia 1 month after undergoing TAVI with a SAPIEN 3 Ultra RESILIA® valve.

## Case presentation

An 83-year-old man with symptomatic severe aortic stenosis and a Society of Thoracic Surgeons (STS) score of 1.85% underwent TAVI via transfemoral access using a 26-mm SAPIEN 3 Ultra RESILIA® valve (Edwards Lifesciences, Irvine, CA, USA). Although the degree of surgical risk was low, the heart team selected TAVI on the basis of the patient’s advanced age and preference. Intraoperative transesophageal echocardiography (TEE) revealed only mild PVL. The valve appeared well seated with good leaflet mobility. He was discharged on postoperative day 10 without any significant complications.

**Fig. 1:**
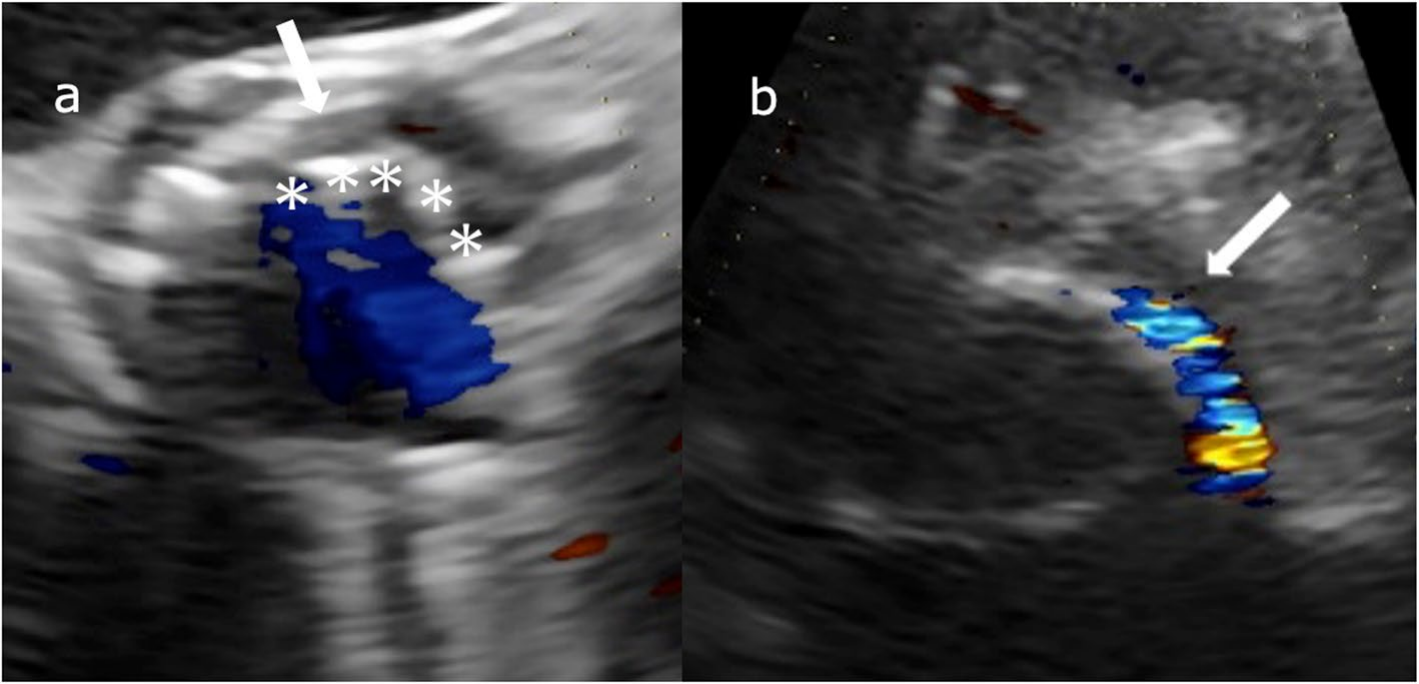
**a** Postoperative transesophageal echocardiography showing a gap between the TAVI valve (asterisk) and the native annulus (arrow), indicating incomplete apposition of the valve. **b** Color Doppler imaging demonstrating mild paravalvular leak (PVL) (arrow)

One month after the procedure, the patient presented with general fatigue. The laboratory findings revealed severe hemolytic anemia, with a hemoglobin level of 9.3 g/dL, a lactate dehydrogenase (LDH) level of 1207 U/L, and a reticulocyte count of 7.3%. The Coombs test was negative, and a peripheral blood smear revealed fragmented red blood cells. Transthoracic echocardiography (TTE) demonstrated mild aortic regurgitation, with a PVL jet originating from the non-coronary cusp (NCC)–left coronary cusp (LCC) commissure, which was directed toward the anterior mitral leaflet. These findings suggested that PVL induced hemolytic anemia following TAVI (Fig. 1).

**Fig. 2:**
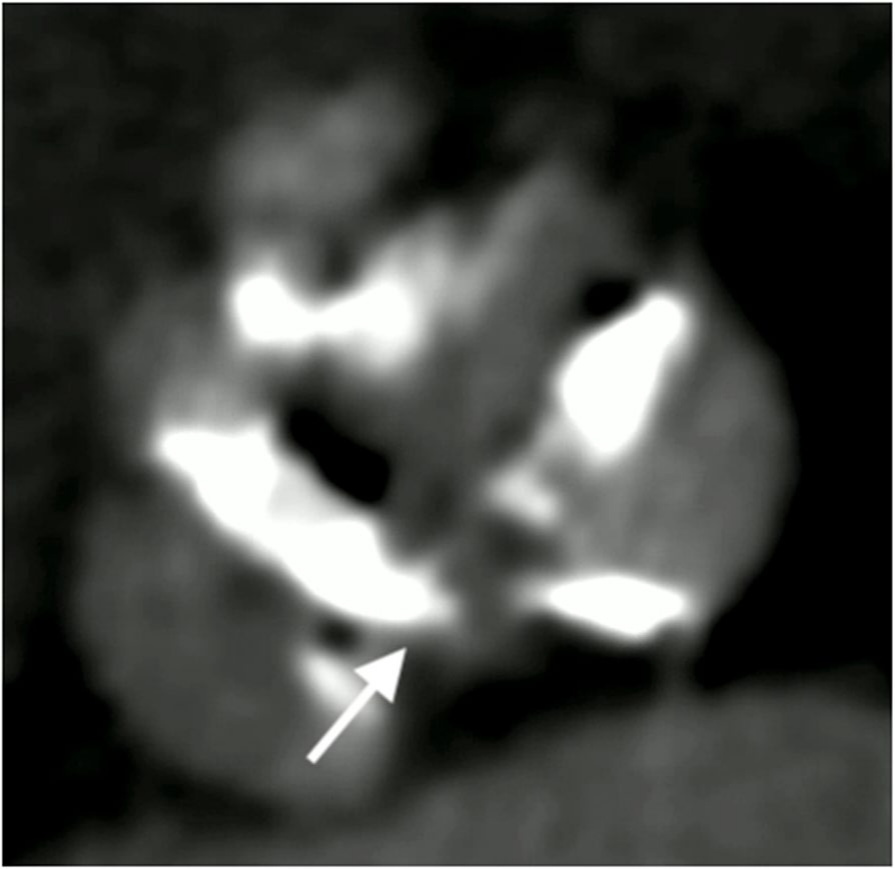
Preoperative computed tomography image showing bulky calcifications on the right coronary cusp (RCC), left coronary cusp (LCC), and non-coronary cusp (NCC). Notably, NCC calcification extends toward the N–L commissure (arrow)

Preoperative computed tomography (CT) revealed bulky calcifications on all three cusps—RCC, LCC, and NCC. In particular, NCC calcification extended toward the NCC–LCC (N–L) commissure (Fig. 2). Given the worsening anemia and ongoing hemolysis, surgical explantation of the transcatheter valve and SAVR were indicated.

**Fig. 3:**
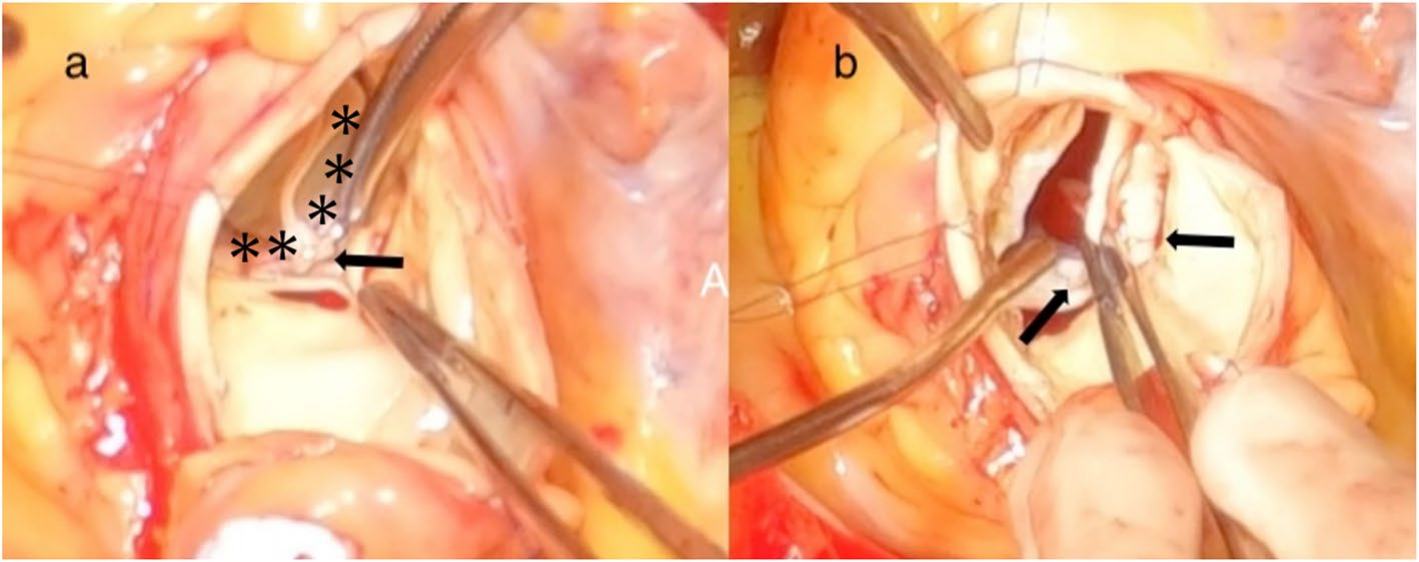
**a** Intraoperative photograph before TAVI valve (asterisk) explantation showing a visible gap at the N–L commissure (arrow). **b** After TAVI valve removal, severe calcifications on the NCC and LCC are evident, which are likely responsible for preventing complete valve expansion at the N–L commissure (arrow)

Cardiopulmonary bypass was established through a median sternotomy under general anesthesia. The ascending aorta was incised approximately 2.5 cm above the transcatheter valve. Intraoperative inspection revealed a gap between the valve frame and the annulus at the N–L commissure (Fig. 3a). The TAVI valve was carefully explanted with minimal adhesion to the surrounding tissue. Severe calcifications were noted on the NCC and LCC, which were believed to have compromised full expansion and apposition of the valve at the N–L commissure (Fig. 3b).

The native aortic valve leaflets and annular calcification were excised, and a 21-mm Avalus® bioprosthetic valve (Medtronic, Minneapolis, MN, USA) was implanted. Postoperative TEE confirmed good valve function with no residual PVL.

Following surgery, the patient’s hemolysis resolved, and he was discharged in stable condition. Although the patient developed mild pleural effusion during follow-up, no major complications were noted.

## Discussion and conclusions

Hemolytic anemia following TAVI is an uncommon but potentially serious complication that is often associated with PVL. While mild PVL is generally considered clinically insignificant, recent evidence suggests that even mild leaks can, in certain anatomical or procedural contexts, lead to significant hemolysis [5, 6].

In a multicenter Japanese study by Ishizu et al., hemolytic anemia developed in 1.7% of patients who underwent TAVI with the SAPIEN 3 Ultra RESILIA® valve [4]. The incidence was significantly greater (33.3%) in cases where undersized valves were implanted and mild or greater PVL was present.

In our case, the patient developed severe hemolytic anemia within 1 month of TAVI, despite only mild PVL observed via echocardiography. Preoperative CT imaging revealed extensive calcification of the aortic cusps, particularly at the N–L commissure. Intraoperative findings confirmed a gap at this location caused by dense calcification that impeded full apposition of the valve stent. These anatomical features likely created high-velocity, eccentric regurgitant flow jets, contributing to red blood cell fragmentation.

In retrospect, the use of a self-expanding valve such as the Evolut™ may have provided better conformability and sealing in this heavily calcified anatomy, especially at the commissural level. Given the supra-annular design and radial force distribution of the Evolut™ valve, it may have mitigated the risk of incomplete apposition. Therefore, individualized valve selection based on preprocedural imaging should be emphasized, particularly in cases with asymmetric or bulky annular calcification.

Various management strategies have been proposed for PVL-induced hemolysis after TAVI, including balloon aortic valvuloplasty (BAV), percutaneous PVL closure using vascular plugs, repeat TAVI, and surgical explantation [9, 10]. Although BAVs may reduce the PVL in select cases, they carry risks such as embolic stroke or annular rupture, especially in patients with bulky calcification [7]. Similarly, percutaneous closure has variable outcomes and is associated with procedural complications, including device embolization, residual leakage, and cardiac tamponade [9]. Moreover, in Japan, percutaneous plug closure remains an off-label and self-funded procedure, limiting its widespread use.

In the present case, the anatomical location and severity of calcification, combined with rapid clinical deterioration, prompted the heart team to opt for surgical explantation. Early explantation, performed within 1 month of the initial TAVI, facilitated relatively easy removal of the prosthesis due to minimal adhesion. Recent data from Fukuhara et al. indicate that surgical explantation in low-risk patients may still result in considerable morbidity and mortality. Delayed surgical intervention—particularly following unsuccessful or suboptimal non-surgical treatments such as repeat TAVR—has been associated with poorer outcomes, highlighting the importance of early and decisive surgical referral when valve dysfunction is identified [3].

With the expanding of TAVI indications to include younger and lower-risk populations, long-term outcomes and the potential need for reintervention have become increasingly relevant. In a retrospective propensity score-matched analysis, patients with low STS scores who underwent SAVR demonstrated superior 5-year outcomes than those who underwent TAVI, despite similar short-term results [11]. Our case underscores that even in low-risk patients, TAVI may result in unanticipated complications necessitating reoperation, thereby negating the perceived benefits of the initial less invasive approach.

In summary, this case highlights the importance of careful preprocedural anatomical evaluation, especially in patients with heavy calcification, even if they are deemed low risk. A mild PVL should not be underestimated, particularly when it occurs at locations prone to eccentric jet formation. Early identification and intervention are essential to prevent the progression of hemolytic anemia, and surgical explantation remains a definitive option in selected cases.

## Data Availability

All the data generated or analyzed during this study are included in this published article.

## Abbreviations

TAVI: Transcatheter aortic valve implantation
SAVR: Surgical aortic valve replacement
PVL: Paravalvular leak
TEE: Transesophageal echocardiography
TTE: Transthoracic echocardiography
LDH: Lactate dehydrogenase
CT: Computed tomography
NCC: Non-coronary cusp
LCC: Left coronary cusp
N–L: NCC–LCC
